# Screening for Health-Promoting Fatty Acids in Ascidians and Seaweeds Grown under the Influence of Fish Farming Activities

**DOI:** 10.3390/md19080469

**Published:** 2021-08-22

**Authors:** Luísa Marques, Maria Rosário Domingues, Elisabete da Costa, Maria Helena Abreu, Ana Isabel Lillebø, Ricardo Calado

**Affiliations:** 1 ECOMARE, CESAM—Centre for Environmental and Marine Studies, Department of Biology, University of Aveiro, Santiago University Campus, 3810-193 Aveiro, Portugal; lillebo@ua.pt; 2 CESAM—Centre for Environmental and Marine Studies, Department of Chemistry, University of Aveiro, Santiago University Campus, 3810-193 Aveiro, Portugal; mrd@ua.pt (M.R.D.); elisabetecosta@ua.pt (E.d.C.); 3 Mass Spectrometry Centre, LAQV-REQUIMTE, Department of Chemistry, University of Aveiro, Santiago University Campus, 3810-193 Aveiro, Portugal; 4 ALGAplus—Production and Trading of Seaweed and Derived Products Lda., 3830-196 Ílhavo, Portugal; helena.abreu@algaplus.pt

**Keywords:** aquafeeds, EPA, DHA, *n*-3/*n*-6 ratio, *n*-3 PUFA, IMTA

## Abstract

The present study aimed to contrast the fatty acid (FA) profile of ascidians (Ascidiacea) and seaweeds (sea lettuce, *Ulva* spp. and bladderwrack, *Fucus* sp.) occurring in a coastal lagoon with versus without the influence of organic-rich effluents from fish farming activities. Our results revealed that ascidians and seaweeds from these contrasting environments displayed significant differences in their FA profiles. The *n*-3/*n*-6 ratio of Ascidiacea was lower under the influence of fish farming conditions, likely a consequence of the growing level of terrestrial-based ingredients rich on *n*-6 FA used in the formulation of aquafeeds. Unsurprisingly, these specimens also displayed significantly higher levels of 18:1(*n*-7+*n*-9) and 18:2*n*-6, as these combined accounted for more than 50% of the total pool of FAs present in formulated aquafeeds. The dissimilarities recorded in the FAs of seaweeds from these different environments were less marked (≈5%), with these being more pronounced in the FA classes of the brown seaweed *Fucus* sp. (namely PUFA). Overall, even under the influence of organic-rich effluents from fish farming activities, ascidians and seaweeds are a valuable source of health-promoting FAs, which confirms their potential for sustainable farming practices, such as integrated multi-trophic aquaculture.

## 1. Introduction

Marine organisms are commonly perceived as a rich source of *n*-3 fatty acids (FA) [1,2,3,4] whose consumption ensures health-promoting benefits against cardiovascular and neurological diseases. Additionally, consumers also acknowledge the anti-inflammatory, anti-coagulation, anti-oxidative properties (among others) of *n*-3 FA originating from seafood, making them paramount for human nutrition [5,6,7,8]. As a result of the fast-growing trend of the world population [8,9] and the high request for nutritious and healthy marine food [1,10,11], aquaculture activities are facing a major challenge in recent years to keep up with an ever-growing demand. Proportionally, there is also a growing focus on the improvement of aquaculture efficiency, as well as the promotion of environmentally and financially sustainable practices [12,13,14,15]. As an example of this ongoing effort, one can refer to the reduction of the levels of marine-based ingredients, such as fishmeal and fish oil, in the formulation of aquafeeds for marine species aquaculture (namely finfish and shrimp) [11,16]. Indeed, a growing proportion of marine-based ingredients have been partially replaced by land-based ingredients (e.g., wheat, soy, corn) [17,18,19] and oils (e.g., palm oil, soybean oil, sunflower oil) [20,21]. Nonetheless, aquafeeds for marine species production still include marine-based ingredients to achieve desirable FA profiles [22]. These marine-based ingredients, particularly fish oil, are a source of essential FAs, such as *n*-3 long-chain polyunsaturated FAs (PUFA) 20:5*n*-3 eicosapentaenoic acid (EPA), and 22:6*n*-3 docosahexaenoic acid (DHA), which are paramount to ensure the healthy development of species being farmed, and as such, safeguard that these remain a valid source of these important nutrients in human diets [23,24]. Consequently, the aquaculture industry has evolved to develop productive frameworks that target the co-production of extractive species that impair the loss of valuable nutrients (such as *n*-3 long-chain PUFA); this approach has been termed integrated multi-trophic aquaculture (IMTA) and has gained a growing interest in the scientific community [25,26,27,28]. These productive systems benefit from the simultaneous farming of species occupying different trophic levels to sequester, recycle and remove excess nutrients originating from uneaten and undigested feed, as well as excretion products [29] present in aquaculture effluents that shape the biochemical content of co-farmed species [10]. Extractive species produced under organic-rich effluents (Org) are responsive to their surrounding environment and experience more or less pronounced shifts in their biochemical composition [2,30,31]. Consequently, FA analysis has become an excellent tool to trace the biochemical fingerprint of aquaculture effluents in aquatic environments and their species [32,33].

Ascidians are marine filter-feeders commonly investigated for marine natural products development, such as anti-cancer and anti-malarial drugs [34]. Knowledge on ascidians’ FA profiling is still poorly explored. However, some studies have already confirmed that ascidians present a high *n*-3/*n*-6 ratio [3,35] and high values of EPA and DHA [36], establishing ascidians as a potential new bioresource for *n*-3 fatty acids-rich marine lipids [3,37,38]. Hassanzadeh [38] concluded that the FA profile of ascidians presented similar values to that of fish oil and, therefore, considered ascidians as a good alternative for fish oil in the formulation of aquafeeds. Additionally, ascidian’s biomass may even successfully replace fishmeal in the formulation of aquafeeds [39,40].

The use of seaweeds has been thoroughly explored in IMTA systems [26,41,42]. Seaweed production under this productive framework is receiving growing attention for mass production given their nutritional value and profile in natural bioactive metabolites (particularly with antioxidant properties) [41,43]. Similar to ascidians, seaweeds are considered an important source of *n*-3 long-chain PUFA, especially α-linolenic acid (ALA; 18:3*n*-3) and EPA [4,44], with their potential as ingredients for aquafeed formulations also being increasingly acknowledged [45]. Although the lipid content in seaweed is relatively low (1.27% to 9.13%) [46], these organisms feature high *n*-3/*n*-6 ratios, making them an appealing source of a valuable source of essential FA in health-promoting diets [47]. 

The present study aimed to compare the FA profile of ascidians (Ascidiacea) and seaweeds (sea lettuce, *Ulva* spp. and bladderwrack, *Fucus* sp.) sampled in a coastal lagoon with versus without the influence of organic-rich effluents from fish farming activities. Additionally, the FA profile of ascidians is also contrasted with that of the most commercially used fish aquafeed employed in the studied location to investigate whether these filter-feeding marine organisms somehow mimicked the FA profile of those aquafeeds when grown under the influence of organic-rich effluents originating from fish farms. 

## 2. Results

### 2.1. Ascidiacea

The FA profile of Ascidiacea revealed a total of 42 different FA (Supplementary Information Table S1). Nonetheless, 4 FAs alone represented more than 50% of the total pool of FAs (Table 1).

**Figure 1:**
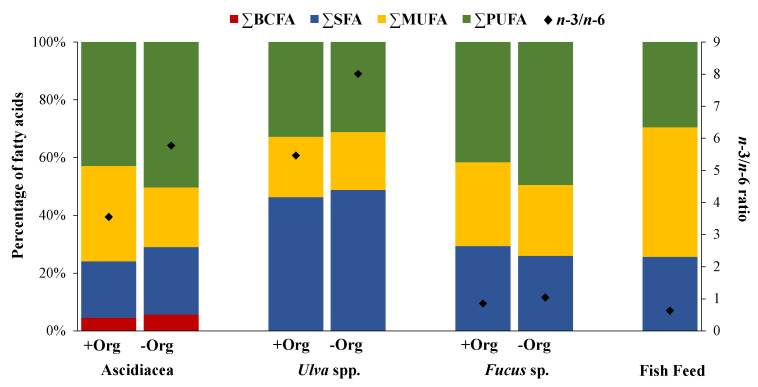
Fatty acid classes expressed as a percentage of the total pool of fatty acids (only values above 1% were considered) of ascidians (Ascidiacea) and seaweeds (sea lettuce, *Ulva* spp. and bladderwrack, *Fucus* sp.) sampled in locations with versus without the influence of organic-rich effluents from fish farming activities (+Org or −Org, respectively), as well as the formulated fish feed (FF) most commonly supplied in fish farming. BCFA: branched fatty acids, SFA: saturated fatty acids, MUFA: monounsaturated fatty acids, PUFA: polyunsaturated fatty acids.

PERMANOVA test revealed the existence of significant differences in the FA profiles (*p* = 0.006) and FA classes (*p* = 0.011) of Ascidiacea from the two locations surveyed (Table 2). Furthermore, statistical differences were also recorded between all FA classes (Table 1). Concerning the *n*-3/*n*-6 ratio, significant differences were detected between both sampling locations (*p* = 0.002) (Table 1), with higher values being recorded for Ascidiacea sampled at −Org (5.77) (Figure 1). In general, all FAs presented a higher relative abundance at –Org, with the exception of FA octadecenoic acid 18:1(*n*-7+*n*-9), 18:2, 18:2*n*-6, and 20:1*n*-9, which displayed higher abundances at +Org. The FAs EPA and DHA were the two most well-represented FAs (17.8% for +Org and 20.4% for −Org; 8.8% for +Org and 11.9% for −Org, respectively) (Table 1). Furthermore, the relative abundance of FAs 18:1(*n*-7+*n*-9), 18:2*n*-6, and DHA differed significantly between the two locations (Table 1). 

Branched FAs (BCFA) represented the least abundant FA class identified in specimens sampled from both locations (4.6% for +Org; 5.5% for −Org) (Figure 1). Saturated FAs (SFA) and PUFA demonstrated higher values in specimens from −Org (22.3% and 48.5%, respectively). In addition, monounsaturated FAs (MUFA) values were higher at +Org (33% for +Org and 20.7% for −Org) (Figure 1). Similarity Percentage Species Contributions (SIMPER) analysis (Table 3A) showed that the FA profiles of Ascidiacea originating from the two locations displayed an average dissimilarity of 10.6%, with more than 50% cumulative dissimilarities being explained by the following FAs: eicosenoic acid 20:1*n*-9, 18:1(*n*-7+*n*-9), linoleic acid—LA 18:2*n*-6, and stearidonic acid—SDA 18:4*n*-3.

### 2.2. Seaweeds

**Figure 2:**
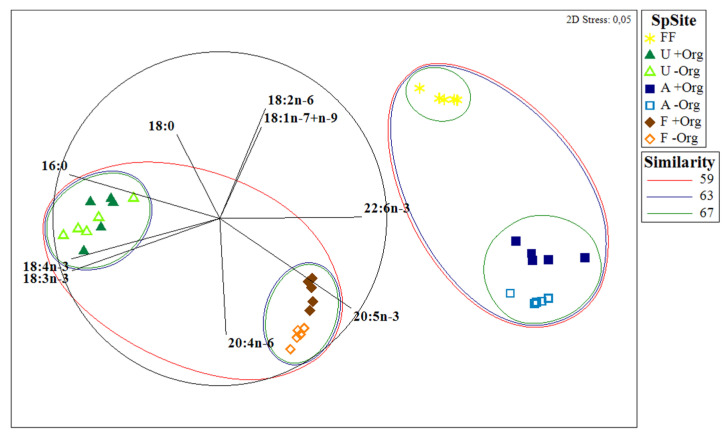
Multidimensional scaling (MDS) ordination plot comparing the fatty acid profiles between specimens of ascidians (Ascidiacea) (A) and seaweeds (sea lettuce, *Ulva* spp. (U) and bladderwrack, *Fucus* sp. (F)) sampled in locations with versus without the influence of organic-rich effluents from fish farming activities (+Org or −Org, respectively) and the formulated fish feed (FF) most commonly supplied in fish farming activities in the study location.

A total of 17 and 24 different FAs were identified for *Ulva* spp. and *Fucus* sp., respectively (Supplementary Information Table S1) (Table 1). The FAs palmitic acid 16:0 and 18:1(*n*-7+*n*-9) were dominant in both seaweeds (37.7% for +Org and 38.1% for −Org; 15.2% for +Org and 15.2% for −Org, respectively). However, some contrasts worth highlighting were also recorded, such as the relative abundance of arachidonic acid (AA) 20:4*n*-6 and EPA in *Fucus* sp. (14.1% for +Org and 15.0% for −Org; 7.7% for +Org and 10.0% for −Org; respectively) that were either not detected or present at trace levels (respectively) in *Ulva* spp. Statistically significant differences were detected in 18:3*n*-3 for *Ulva* spp. (*p* = 0.025), while for *Fucus* sp. FAs 18:1(*n*-7+*n*-9), 18:2*n*-6, 18:3*n*-3, and EPA all differed significantly (*p* = 0.003, *p* = 0.013, *p* < 0.001, *p* < 0.001, respectively). PERMANOVA test showed statistical differences in the mean FA profiles of seaweeds originating from the two sampling locations (*p* = 0.021 for *Ulva* spp.; *p* = 0.013 for *Fucus* sp.), yet only significant differences were seen in the FA classes of *Fucus* sp. (*p* = 0.013) (Table 2), with significant differences being recorded between MUFA and PUFA (*p* = 0.005, *p* < 0.001, respectively) of specimens of this brown seaweed originating from the two sampling locations (Table 1). The *n*-3/*n*-6 ratio also exhibited significant differences between both sampling locations (*p* < 0.001 for *Ulva* spp., *p* < 0.001 for *Fucus* sp.) (Table 1), with higher values being recorded for seaweeds at −Org. The prevailing FA class in *Ulva* spp. was SFA (46.3% for +Org and 48.8% for −Org) (Figure 1), while PUFA registered higher values for *Fucus* sp. (41.6% for +Org; 49.4% for −Org). The non-metric multidimensional scaling (MDS) plot (Figure 2) revealed a distinct separation between the two seaweeds and the two sampling sites, with similarity values of 59% grouping both FA profiles. 

SIMPER analysis (Table 3A) revealed that the FA profiles of *Ulva* spp. and *Fucus* sp. display comparable values of dissimilarities between +Org and −Org (5.29% and 5.48%, respectively), with FA 18:0 contributing the most for such differences.

### 2.3. Fish Feed

A total of 26 FAs were identified in fish feed (Supplementary Information Table S1) (Table 1). MUFA was the most abundant FA class for fish feed (44.8%) (Figure 1) with a major contribution of FA 18:1(*n*-7+*n*-9) (36.0%) (Table 1). SFA and PUFA presented similar values (25.7% and 29.5%, respectively). The *n*-3/*n*-6 ratio obtained was 0.63, indicating higher amounts of *n*-6 FAs. The MDS plot (Figure 2) revealed that the FA profile of fish feed is more similar to the FA profile of Ascidiacea from +Org than from −Org. SIMPER analysis of the FA profiles of fish feed and Ascidiacea (Table 3B) revealed higher dissimilarities with specimens originating from −Org. For Ascidiacea, EPA was the main responsible for such differences.

## 3. Discussion

To the authors’ best knowledge, the present study is the first approach reported in the scientific literature to screen for health-promoting FAs in ascidians grown under the influence of fish farming organic-rich effluents. From the total pool of FA identified in Ascidiacea (42 FAs), only 4 of these biomolecules (16:0, 18:1(*n*-7+*n*-9), EPA, and DHA) represented average values above 10% of the total pool of FA. These findings share similarities with those reported from previous works screening the FA profile of ascidians [37,48,49,50]. The FAs 18:1(*n*-7+*n*-9) and 18:2*n*-6 also displayed higher values in +Org, near twice the ones recorded for −Org. Considering that these FAs accounted for 53% of the fish aquafeed FA pool, it is likely that ascidians may selectively retain these FAs in their tissues. The higher levels of *n*-3 FAs present in the −Org resulted in a higher *n*-3/*n*-6 ratio, with FAs 18:4*n*-3, EPA, and DHA being the main contributors to this trend. This finding is consistent with Monmai et al. [35], as these authors verified that in the edible ascidian *Halocynthia aurantium n*-3 FAs were present in much higher levels than *n*-6 FAs. Likewise, Zhao and Li [37] documented that tunics and inner body tissues of ascidians *Halocynthia roretzi*, *Styela plicata*, *Ascidia* sp. and *Ciona intestinalis* presented higher levels of *n*-3 FAs. 

*Ulva* spp. and *Fucus* sp. presented some similarities in their FA profiles, with 16:0 and 18:1(*n*-7+*n*-9) displaying the highest relative abundances in the total pool of FAs recorded in both locations. This finding is in line with previous studies [51,52,53]. Our results on the profiling of unsaturated FAs (MUFA+PUFA) are fully aligned with those reported by Herbreteau et al. [54], who reported the FA composition of five species of seaweeds and verified that unsaturated FAs accounted for more than 50% of the total pool of FAs, with this proportion reaching up to 75% for *Fucus* sp. Silva et al. [55] focused on ten brown seaweeds also verifying important amounts of unsaturated FAs. In addition, our study recorded 46% to 49% of SFA in *Ulva* spp., unlike Lopes et al. [4] who have reported about half of these values for the same seaweed species (≈24%). Yet, the values of FA classes reported for *Fucus* sp. by Lopes et al. [4] are very much in line with the ones reported in the present work. Several studies [4,55,56] have mentioned that despite lipid content representing a minor fraction of seaweeds, it features levels of *n*-3 PUFAs worth being investigated. Our results validated the presence of EPA in *Fucus* sp., but not DHA, and no traces of either of these FAs were detected in *Ulva* spp. These latter values correlate fairly well with Pereira et al., [57] with *Ulva* spp. also presenting higher proportions of FA 18:3*n*-3, and thus, further supporting the idea that seaweeds do display an *n*-3/*n*-6 “healthy” ratio.

Several studies [1,5,6,30,58] have reported an increase in the use of *n*-6 PUFA-rich land-based ingredients and oils in aquafeed formulations sometimes leading to an inverted *n*-3/*n*-6 ratio in fish aquafeeds. Under organic-rich effluents, the biochemical profile of extractive species will most likely be shaped by the prevalence of these ingredients [16]. However, the availability of natural nutrients [59], sampling location, and season [55], amongst other factors, must be taken into consideration when profiling the FAs of marine species, as they too can modulate their biochemical profile and findings being reported results must be interpreted with care. Kim et al. [52] demonstrated how temperature, salinity, light, and nitrogen levels influence the level and profile of lipids present in the brown seaweed *Fucus serratus*. Similar findings were reported by Glencross [23] who emphasized how the hydrological source is a primary factor weighing in on the differences in FA requirements. This trend can extend to a multitude of marine organisms of interest for production under an IMTA framework, such as polychaetes [60,61], isopods [62], bivalves [63,64], and several fish species [65].

In conclusion, the present study demonstrated that Ascidiacea presented high values of EPA (17.8% in +Org; 20.4% in −Org) and DHA (8.8% in +Org; 11.9% in −Org) and can be considered as a potential new bioresource for *n*-3 long-chain FAs. The organic-rich effluent originating from fish farming systems can indeed shape the lipid profile of extractive species being employed in IMTA frameworks, whether as a consequence of direct consumption of available organic nutrients in dissolved and particulate form, as in the case of ascidians, or indirectly from de novo FA synthesis as in the case of seaweeds uptaking dissolved inorganic nutrients. The use of extractive species to maximize the use of ingredients present in formulated aquafeeds employed to farm marine finfish and shrimp can be considered as a pathway towards more sustainable and efficient aquaculture practices and have the potential to generate biomass with the potential to deliver important biomolecules for multiple biotechnological applications [66]. Our findings clearly point towards the need to further investigate the biochemical profile, particularly the FA profile of extractive species used in IMTA systems, as an approach to sequester valuable health-promoting FAs that will otherwise be lost to the aquatic environment through the effluents of fish farms.

## 4. Materials and Methods

### 4.1. Study Areas

**Figure 3:**
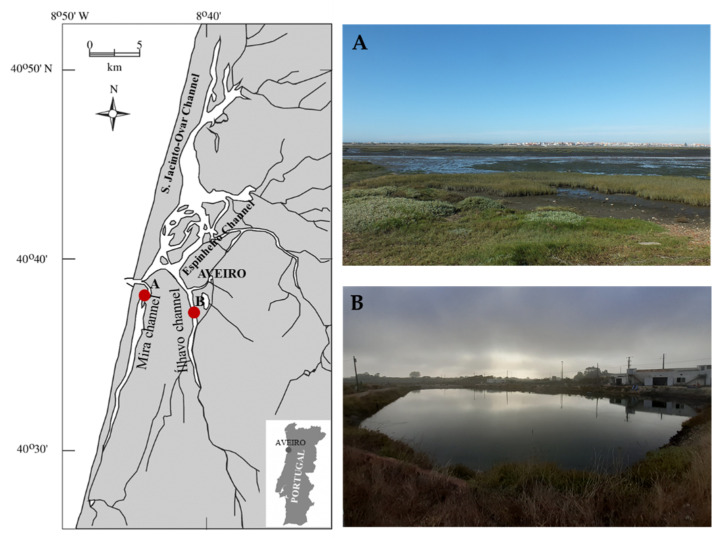
Sampling locations at Ria de Aveiro coastal lagoon (Portugal): (**A**) located in Mira channel (40°36’51” N, 8°44’25” W) and without the influence of organic-rich effluents from fish farming activities (−Org); and (**B**) located at a land-based semi-intensive fish farm (40°36’43’’ N, 8°40’43’’ W) supplied by Ílhavo channel’s waters employing an IMTA framework where seaweeds are produced in tanks supplied with organic-rich waters from earthen ponds stocked with fish (+Org).

Ria de Aveiro is a shallow coastal lagoon in the west margin of mainland Portugal that inholds the Vouga river estuary and presents a complex and irregular geometry. This coastal lagoon has four main channels emerging from the sea entrance: S. Jacinto-Ovar, Espinheiro, Ílhavo, and Mira channel (Figure 3). The first sampling location surveyed was located at Mira channel (40°36’51” N, 8°44’25” W) without the influence of organic-rich effluents from fish farming activities and is herein referred as −Org. The second sampling location surveyed was located at a land-based semi-intensive fish farm (40°36’43’’ N, 8°40’43’’ W) supplied by Ílhavo channel’s waters. An IMTA framework is employed in this location, on which European seabass and Gilthead seabream are produced in earthen ponds and seaweeds are produced in tanks supplied with organic-rich waters from these earthen ponds. This location will be referred to as +Org. Both channels of this coastal lagoon present strong salinity gradients with very low values at their upper reaches. Salinity, temperature, dissolved oxygen, and pH were registered in situ at the time of sampling. Environmental parameters are summarized as Supplementary Information (Table S2).

### 4.2. Sample Collection

#### 4.2.1. Ascidiacea

Ascidians were collected manually from both locations described above. The taxonomic identification of ascidians is complex, and producers are unable to readily sort them by species, namely if they target the production of small sized specimens (when key diagnosing morphological features are incipient). While *Styela plicata* and *Ciona intestinalis* were certainly present among the ascidians collected, it is not impossible to rule out the presence of other species without using molecular tools (e.g., DNA barcodes) or taxonomic identification by experts. As such, ascidians were pooled into composite samples and will be simply termed as Ascidiacea. All specimens were left to depurate for 48 h after being sampled, in order to safeguard that their guts were emptied and, as such, avoid any bias on their FA profile from dietary prey. All specimens were depurated using filtered seawater (GFFC, glass microfiber filter 1.2 µm, Ø47 mm) from their sampling locations. After depuration all specimens were washed thoroughly using tap water to eliminate any impurities and all five composite samples of 3 individuals each (of similar sizes) were selected per sampling location. All samples were freeze-dried and stored at -20 °C. Prior to FA analysis, samples were grounded into a fine powder using a mortar and pestle.

#### 4.2.2. Seaweeds

Specimens from the genus *Ulva* (Chlorophyceae) and *Fucus* (Phaeophyceae) were collected from the same locations as ascidians (−Org and +Org). As already detailed above for ascidians, more than one species of *Ulva* can be present in one or both of the sampling locations surveyed in the present work. As such, all collected sea lettuce samples were termed as *Ulva* spp. Concerning the samples of bladderwrack collected in the present work, all specimens of this brown seaweed could be easily identified to the species level (*Fucus vesiculosus*), but to keep consistency with the identification level of the green seaweed, it will be addressed as one species of the genus *Fucus*. All seaweeds were washed using tap water to eliminate impurities and excess water was dried from samples. Five composite samples of five seaweeds each were separated by species and location, freeze-dried and stored at -20 °C. As for ascidians, seaweeds biomass was also grounded into a fine powder using a mortar and pestle.

#### 4.2.3. Fish Feed

The FA profile of the formulated fish feed (Standard Orange 4; SORGAL, Sociedade de óleos e rações, SA) supplied at the fish farm operating under an IMTA framework was determined using 250 mg of feed per each of the five replicates analyzed (Table S3 for detailed composition). All storage and processing of these samples prior to FA analysis were identical to those described above for ascidians and seaweeds.

### 4.3. Total Lipid Extraction

Lipid extraction was performed by adding 3.75 mL of a mixture of methanol/chloroform (2:1, *v*/*v*) to 150 mg of ascidians and 250 mg of seaweeds (five biological replicates per biological matrix tested) in a glass test tube with a Teflon-lined screw cap. Samples were then homogenized and incubated in ice on a rocking platform shaker (Stuart Scientific STR6, Bibby, UK) for 2 h and 30 min. The mixture was centrifuged at 2000 rpm for 10 min., and after the organic phase was collected. The biomass residue was re-extracted two times with 2 mL of methanol and 1 mL of chloroform. Afterward, water was added (2.3 mL) to the total collected organic phase, centrifuged at 2000 rpm for 10 min and the organic (lower) phase was recovered. Solvents were dried under a stream of nitrogen gas. Total lipid extract was estimated by gravimetry. Lipid extracts were stored in dark vials and stored at −20 °C before analysis by gas chromatography-mass spectrometry (GC–MS). Reagents were purchased from Fisher Scientific Ltd. (Loughborough, UK). All other reagents were purchased from major commercial suppliers. Milli-Q water (Synergy, Millipore Corporation, Billerica, MA, USA) was used.

### 4.4. Fatty Acid Profiling

Fatty acid methyl esters (FAMEs) were prepared using a methanolic solution of potassium hydroxide (2.0 M) as described by Melo et al. [67]. Subsequently, 2.0 μL of a hexane solution containing FAMEs were analyzed by GC–MS on an Agilent Technologies 6890 N Network (Santa Clara, CA, USA) equipped with a DB–FFAP column. The column was 30 m long, had 0.32 mm of internal diameter, and a film thickness of 0.25 μm (123-3232, J&W Scientific, Folsom, CA, USA). The GC equipment was connected to a Mass Selective Detector (Agilent 5973 Network) operating with an electron impact mode at 70 eV and scanning the range m/z 50–550 in a 1 s cycle in a full scan mode acquisition. The carrier gas Helium was used at a flow rate of 1.4 mL min^−1^. The elution relied on an increasing temperature gradient: 80 °C for 3 min, a linear increase to 160 °C at 25 °C min^−1^, followed by a linear increase at 2 °C min^−1^ to 210 °C, then at 30 °C min^−1^ to 250 °C, standing at 250 °C for 10 min Identification of FAs was performed considering retention times and mass spectrometry spectra of FA standards (Supelco 37 Component Fame Mix, Sigma-Aldrich, St. Louis, MO, USA), as well as through mass spectrum comparison with those in Wiley 275 library and AOCS Lipid Library. The relative amounts of FAs were calculated by the percent area method with proper normalization, considering the sum of all areas of the identified FAs. The results were expressed as means ± SD.

### 4.5. Statistical Analysis

Data from FA profiles were square-rooted transformed, and a Bray-Curtis matrix was assembled. A one-way PERMANOVA was used to test for differences between the FA profiles (for both all individual FAs, as well as FA classes) of Ascidiacea and seaweeds originating from +Org and −Org, with “sampling location” being used as a fixed factor. The statistical significance of variance components was tested using 999 permutations of unrestricted permutations of data, with an a priori chose significance level of α = 0.05. Individual differences in the relative abundance of FA (whose values recorded > 5% of the total pool of FA in at least one of the biological matrices surveyed), FA classes, ∑*n*-3, ∑*n*-6, and the ∑*n*-3/∑*n*-6 ratio from +Org and −Org were compared by either a t-test or the non-parametric Mann-Whitney U rank comparisons if samples were not normally distributed. A MDS was used to graphically visualize overall patterns and relationships between the different biological matrices survey. A SIMPER analysis was used to determine which FAs contributed the most to similarities and dissimilarities within Ascidiacea and seaweeds, at a cut-off of 50%. All analyses were performed using the PRIMER 6 + PERMANOVA© software (software package from Plymouth Marine Laboratory, Plymouth, UK).

## Figures and Tables

**Table 1:** Fatty acid profile of ascidians (Ascidiacea) and seaweeds (sea lettuce, *Ulva* spp. and bladderwrack, *Fucus* sp.) sampled in locations with versus without the influence of organic-rich effluents from fish farming activities (+Org or −Org, respectively), as well as the formulated fish feed (FF) most commonly supplied in fish farming activities in the study location. Values are expressed as a percentage of the total pool of fatty acids and are averages of five replicates (*n* = 5) ± SD. Only fatty acids accounting for at least 5% of the total pool of fatty acids in one of the biological matrices surveyed are presented. SFA: saturated fatty acids, MUFA: monounsaturated fatty acids, PUFA: polyunsaturated fatty acids.

	Ascidiacea		*Ulva* spp.		*Fucus* sp.		Fish Feed
	+Org	−Org	+Org	−Org	+Org	−Org	
14:0	0.94 ± 0.17	1.45 ± 0.12	0.68 ± 0.22	0.64 ± 0.17	8.04 ± 0.64	8.47 ± 0.27	1.53 ± 0.35
16:0	11.50 ± 1.31	12.56 ± 0.67	37.74 ± 1.14	38.05 ± 1.86	16.17 ± 1.29	15.03 ± 0.62	17.25 ± 0.68
16:1*n*-9	5.78 ± 0.62	5.37 ± 0.29	3.33 ± 0.27	2.67 ± 0.27	0.25 ± 0.04	0.29 ± 0.03	3.62 ± 0.18
16:4*n*-3	n.d	n.d	5.18 ± 0.33	4.27 ± 0.67	0.59 ± 0.06	0.59 ± 0.05	n.d
18:0	4.87 ± 1.23	5.89 ± 0.53	6.58 ± 3.99	8.70 ± 2.29	4.34 ± 1.61	1.77 ± 0.16	6.51 ± 1.09
18:1*n*-7+*n*-9	20.27 ± 1.80	11.98 ± 0.95 **	15.23 ± 1.21	15.19 ± 1.22	26.50 ± 2.28	21.34 ± 1.51 *	35.97 ± 0.43
18:2*n*-6	5.85 ± 1.62	2.26 ± 0.08 *	4.41 ± 0.19	2.74 ± 0.41	6.82 ± 0.38	7.45 ± 0.21 *	16.86 ± 0.19
18:3*n*-3	2.16 ± 0.22	2.38 ± 0.48	8.95 ± 0.70	7.85 ± 0.57 *	6.96 ± 0.41	8.87 ± 0.51 **	2.85 ± 0.07
18:4*n*-3	1.54 ± 0.61	3.61 ± 0.69	9.72 ± 0.65	10.10 ± 0.72	3.70 ± 0.36	5.55 ± 0.62	0.62 ± 0.05
20:4*n*-6	2.43 ± 0.37	3.11 ± 0.27	n.d	n.d	14.08 ± 1.17	15.03 ± 0.22	0.47 ± 0.03
20:5*n*-3	17.77 ± 2.90	20.44 ± 1.00	0.61 ± 0.13	1.25 ± 1.14	7.66 ± 0.74	9.95 ± 0.39 **	2.13 ± 0.11
22:6*n*-3	8.75 ± 1.00	11.85 ± 1.01 **	n.d	n.d	n.d	n.d	4.59 ± 0.32
∑*n*-3	32.03 ± 3.62	40.07 ± 1.54 *	27.35 ± 1.87	27.61 ± 2.30	19.16 ± 1.54	25.24 ± 1.42 **	11.43 ± 0.51
∑*n*-6	9.02 ± 1.25	6.94 ± 0.46 *	5.00 ± 0.24	3.45 ± 0.44 **	22.42 ± 1.59	24.18 ± 0.07 *	18.09 ± 0.23
∑*n*-3/∑*n*-6	3.66 ± 0.98	5.79 ± 0.37 *	5.46 ± 0.25	8.04 ± 0.36 **	0.85 ± 0.03	1.04 ± 0.06 **	0.63 ± 0.03
∑SFA	19.52 ± 2.36	22.39 ±1.00 *	46.30 ± 3.35	48.78 ± 3.37	29.35 ± 3.48	26.02 ± 0.50	25.72 ± 1.42
∑MUFA	32.99 ± 0.92	19.95 ± 1.39 **	20.88 ± 1.62	20.07 ± 1.66	29.07 ± 2.32	24.42 ± 1.48 *	44.77 ± 0.81
∑PUFA	42.81 ± 2.65	48.48 ± 1.80 *	32.82 ± 1.94	31.19 ± 2.73	41.58 ± 3.08	49.43 ± 1.42 **	29.52 ± 0.64

nd: not detected; * *p* < 0.05; ** *p* < 0.001. ∑SFA: 14:0, 15:0, 16:0, 17:0; 18:0, 20:0, 21:0, 22:0, 24:0; ∑MUFA: 15:1, 16:1, 16:1*n*-7, 16:1n-9, 17:1, 17:1*n*-9, 18:1*n*-7+*n*-9, 20:1, 20:1*n*-9, 20:1*n*-7, 22:1*n*-11, 22:1*n*-9, 24:1*n*-9; ∑PUFA: 16:2, 16:2*n*-6, 16:3*n*-3, 16:4*n*-3, 18:2, 18:2*n*-6, 18:3*n*-6, 18:3*n*-3, 18:4*n*-3, 20:2, 20:2*n*-6, 20:3*n*-6, 20:3*n*-3, 20:4*n*-6, 20:4*n*-3, 20:5*n*-3, 22:4, 22:4, 22:5*n*-6, 22:5*n*-3, 22:6*n*-3.

**Table 2:** The results of the permutational multivariate analysis of variance (PERMANOVA) of fatty acids and fatty acid classes of ascidians (Ascidiacea) and seaweeds (sea lettuce, *Ulva* spp. and bladderwrack, *Fucus* sp.) sampled in locations with versus without the influence of organic-rich effluents from fish farming activities (+Org or −Org, respectively). Significant differences were considered at *p* < 0.05 (represented in bold); P(perm): *p*-values based on more than 999 permutations.

	Permanova	
	+Org vs. −Org	
	Fatty Acids	Fatty Acids Classes
**Ascidiacea**	**0.006**	**0.011**
***Ulva* spp.**	**0.021**	0.341
***Fucus* sp.**	**0.013**	**0.013**

**Table 3:** Summary of SIMPER analysis listing the fatty acids that most contributed to discriminate: (**A**) ascidians (Ascidiacea) and seaweeds (sea lettuce, *Ulva* spp. and bladderwrack, *Fucus* sp.) sampled in locations with versus without the influence of organic-rich effluents from fish farming activities (+Org or −Org, respectively); and (**B**) ascidians from +Org or −Org with the formulated fish feed (FF) most commonly supplied in fish farming activities in the study location. Cut-off percentage: 50%.

	Dissimilarity											
**(A)**	**Ascidiacea**				***Ulva* spp.**				***Fucus* sp.**			
	**+Org vs. −Org**				**+Org vs. −Org**				**+Org vs. −Org**			
	**10.62%**				**5.29%**				**5.48%**			
**−Org**		+ORW	−ORW	Contrib%		+Org	−Org	Contrib%		+Org	−Org	Contrib%
**vs.**	20:1*n*-9	2.21	1.03	15.81	18:0	2.48	2.93	23.19	18:0	2.05	1.33	22.78
**+Org**	18:1*n*-7+*n*-9	4.50	3.46	13.92	18:2*n*-6	2.10	1.65	14.23	18:1*n*-7+*n*-9	5.14	4.62	16.65
	18:2*n*-6	2.40	1.50	11.94	22:5*n*-3	1.54	1.91	11.59	18:4*n*-3	1.92	2.35	13.56
	18:4*n*-3	1.22	1.89	8.98	20:5*n*-3	0.78	1.04	9.76				
**(B)**	**Ascidiacea**											
	**+Org vs. FF**				**−Org vs. FF**							
	**31.06%**				**36.35%**							
		+Org	FF	Contrib%		−Org	FF	Contrib%				
**Org**	20:5*n*-3	4.21	1.46	13.91	20:5*n*-3	4.52	1.46	13.33				
**vs.**	18:2*n*-6	2.40	4.11	8.65	18:2*n*-6	1.50	4.11	11.34				
**FF**	20:4*n*-6	1.55	0	7.87	18:1*n*-7+*n*-9	3.46	6.00	11.06				
	18:1*n*-7+*n*-9	4.5	6.00	7.58	18:4*n*-3	1.89	0	8.24				
	22:1*n*-11	0	1.36	7.05	20:4*n*-6	1.76	0	7.68				
	18:4*n*-3	1.22	0	6.18								

## Data Availability

The data presented in this study are included in the corresponding sections throughout the manuscript.

## Supplementary Materials

The following are available online at https://www.mdpi.com/article/10.3390/md19080469/s1, Table S1: Fatty acid profile of ascidians (Ascidiacea) and seaweeds (sea lettuce, *Ulva* spp. and bladderwrack, *Fucus* sp.) sampled in locations with versus without the influence of organic-rich effluents from fish farming activities (+Org or −Org, respectively), as well as the formulated fish feed (FF) most commonly supplied in fish farming activities in the study location. Values are expressed as a percentage of the total pool of fatty acids and are averages of five replicates (n=5) ± SD. BCFA: Branched fatty acids, SFA: saturated fatty acids, MUFA: monounsaturated fatty acids, PUFA: polyunsaturated fatty acids. n.d: not detected; Table S2: Summary of the environmental parameters measured at the time of sampling in locations with versus without the influence of organic-rich waters from fish farming activities (+Org or −Org, respectively). Values are expressed as a percentage and are averages of three replicates (n=6) ± SD; Table S3: Nutritional composition of the formulated fish feed provided to the fish at the fish farming location (+Org).
