# Supplementary material for: Screening for Health-Promoting Fatty Acids in Ascidians and Seaweeds Grown under the Influence of Fish Farming Activities
Source: Mar Drugs. 2021 Aug 22;19(8):469. doi: 10.3390/md19080469 (PMC8400344; doi:10.3390/md19080469)
Supplement: Supplementary file 1 [file marinedrugs-19-00469-s001.zip › marinedrugs-1354873-supplementary.pdf]

Screening for health-promoting fatty acids in ascidians and seaweeds grown under the influence of fish farming activities

Luísa Marques[1*], M. Rosário Domingues[2,3], Elisabete da Costa[2,3], Maria Helena Abreu[4], Ana Isabel Lillebø[1], Ricardo Calado[1,*]

[1] ECOMARE, CESAM - Centre for Environmental and Marine Studies, Department of Biology, University of Aveiro, Santiago University Campus, 3810-193 Aveiro, Portugal; luisa.marques@ua.pt (L.M.); lillebo@ua.pt (A.L.) rjcalado@ua.pt (R.C.)

[2] CESAM - Centre for Environmental and Marine Studies, Department of Chemistry, University of Aveiro, Santiago University Campus, 3810-193 Aveiro, Portugal; mrd@ua.pt (M.R.D.) elisabetecosta@ua.pt (E.d.C.)

[3] Mass Spectrometry Centre, LAQV-REQUIMTE, Department of Chemistry, University of Aveiro, Santiago University Campus, 3810-193 Aveiro, Portugal

[4] ALGAplus - Production and Trading of Seaweed and Derived Products Lda., 3830-196 Ílhavo, Portugal; helena.abreu@algaplus.pt

* Correspondence: luisa.marques@ua.pt; +351 967 676 182 (LM); rjcalado@ua.pt; +351 917 989 010 (RC)

Supplementary Information (SI)

Table SI1: Fatty acid profile of ascidians (Ascidiacea) and seaweeds (sea lettuce, *Ulva* spp. and bladderwrack, *Fucus* sp.) sampled in locations with *versus* without the influence of organic-rich effluents from fish farming activities (+Org or -Org, respectively), as well as the formulated fish feed (FF) most commonly supplied in fish farming activities in the study location. Values are expressed as a percentage of the total pool of fatty acids and are averages of five replicates (n=5) ± SD. BCFA: Branched fatty acids, SFA: saturated fatty acids, MUFA: monounsaturated fatty acids, PUFA: polyunsaturated fatty acids. n.d: not detected.

	**Ascidiacea**		***Ulva* spp.**		***Fucus* sp.**		**Fish Feed**
	+Org	-Org	+Org	-Org	+Org	-Org	
iso 14:0	0.16 ± 0.02	0.09 ± 0.02	n.d	n.d	n.d	n.d	n.d
14:0	0.94 ± 0.17	1.45 ± 0.12	0.68 ± 0.22	0.64 ± 0.17	8.04 ± 0.64	8.47 ± 0.27	1.53 ± 0.35
iso 15:0	1.44 ± 0.20	2.14 ± 0.15	n.d	n.d	n.d	n.d	n.d
anteiso 15:0	0.74 ± 0.13	0.27 ± 0.03	n.d	n.d	n.d	n.d	n.d
15:0	1.07 ± 0.18	0.61 ± 0.06	n.d	n.d	n.d	n.d	n.d
15:1	0.28 ± 0.06	0.34 ± 0.04	n.d	n.d	n.d	n.d	n.d
iso 16:0	0.59 ± 0.07	0.84 ± 0.04	n.d	n.d	n.d	n.d	n.d
anteiso 16:0	0.12 ± 0.03	0.05 ± 0.01	n.d	n.d	n.d	n.d	n.d
16:0	11.50 ± 1.31	12.56 ± 0.67	37.74 ± 1.14	38.05 ± 1.86	16.17 ± 1.29	15.03 ± 0.62	17.25 ± 0.68
16:1	0.36 ± 0.09	0.26 ± 0.05	n.d	n.d	n.d	n.d	n.d
16:1*n*-7	0.22 ± 0.03	0.38 ± 0.03	2.32 ± 0.43	2.21 ± 0.40	1.03 ± 0.04	1.46 ± 0.09	0.34 ± 0.04
16:1*n*-9	5.78 ± 0.62	5.37 ± 0.29	3.33 ± 0.27	2.67 ± 0.27	0.25 ± 0.04	0.29 ± 0.03	3.62 ± 0.18
16:2	n.d	n.d	0.47 ± 0.13	0.13 ± 0.01	n.d	n.d	n.d
16:2*n*-6	0.13 ± 0.03	0.16 ± 0.03	n.d	n.d	0.05 ± 0.01	0.30 ± 0.08	0.10 ± 0.02
16:3*n*-3	n.d	n.d	n.d	n.d	n.d	n.d	n.d
16:4*n*-3	n.d	n.d	5.18 ± 0.33	4.27 ± 0.67	0.59 ± 0.06	0.59 ± 0.05	n.d
iso 17:0	0.69 ± 0.08	1.51 ± 0.11	n.d	n.d	n.d	n.d	n.d
anteiso 17:0	0.45 ± 0.05	0.48 ± 0.02	n.d	n.d	n.d	n.d	n.d
17:0	0.64 ± 0.06	1.23 ± 0.12	n.d	n.d	n.d	n.d	n.d
17:1	0.26 ± 0.06	0.11 ± 0.01	n.d	n.d	n.d	n.d	n.d
17:1*n*-9	0.56 ± 0.10	0.28 ± 0.02	n.d	n.d	n.d	n.d	n.d
methyl-heptadecanoate	0.40 ± 0.09	0.10 ± 0.01	n.d	n.d	n.d	n.d	n.d
18:0	4.87 ± 1.23	5.89 ± 0.53	6.58 ± 3.99	8.70 ± 2.29	4.34 ± 1.61	1.77 ± 0.16	6.51 ± 1.09
18:1*n*-7+*n*-9	20.27 ± 1.80	11.98 ± 0.95	15.23 ± 1.21	15.19 ± 1.22	26.50 ± 2.28	21.34 ± 1.51	35.97 ± 0.43

18:2	1.19 ± 0.09	0.62 ± 0.09	n.d	n.d	n.d	n.d	n.d
18:2*n*-6	5.85 ± 1.62	2.26 ± 0.08	4.41 ± 0.19	2.74 ± 0.41	6.82 ± 0.38	7.45 ± 0.21	16.86 ± 0.19
18:3*n*-6	0.07 ± 0.02	0.29 ± 0.03	0.59 ± 0.07	0.71 ± 0.05	0.48 ± 0.03	0.46 ± 0.02	0.18 ± 0.02
18:3*n*-3	2.16 ± 0.22	2.38 ± 0.48	8.95 ± 0.70	7.85 ± 0.57	6.96 ± 0.41	8.87 ± 0.51	2.85 ± 0.07
18:4*n*-3	1.54 ± 0.61	3.61 ± 0.69	9.72 ± 0.65	10.1 ± 0.72	3.70 ± 0.36	5.55 ± 0.62	0.62 ± 0.05
20:0	0.41 ± 0.04	0.53 ± 0.04	n.d	n.d	0.36 ± 0.03	0.31 ± 0.04	0.27 ± 0.03
20:1	0.37 ± 0.17	0.16 ± 0.04	n.d	n.d	n.d	n.d	n.d
20:1*n*-9	4.90 ± 0.57	1.06 ± 0.08	n.d	n.d	0.31 ± 0.04	0.26 ± 0.03	2.38 ± 0.06
20:1*n*-7	n.d	n.d	n.d	n.d	n.d	n.d	0.22 ± 0.02
20:2	0.20 ± 0.04	0.16 ± 0.01	n.d	n.d	n.d	n.d	n.d
20:2*n*-6	n.d	n.d	n.d	n.d	0.33 ± 0.01	0.31 ± 0.06	0.49 ± 0.03
20:3*n*-6	0.23 ± 0.04	0.35 ± 0.04	n.d	n.d	0.66 ± 0.04	0.64 ± 0.09	n.d
20:3*n*-3	n.d	n.d	n.d	n.d	n.d	n.d	0.18 ± 0.03
20:4*n*-6	2.43 ± 0.37	3.11 ± 0.27	n.d	n.d	14.08 ± 1.17	15.03 ± 0.22	0.47 ± 0.03
20:4*n*-3	0.74 ± 0.06	0.76 ± 0.17	0.51 ± 0.07	0.50 ± 0.03	0.23 ± 0.02	0.28 ± 0.05	0.36 ± 0.03
20:5*n*-3	17.77 ± 2.90	20.44 ± 1.00	0.61 ± 0.13	1.25 ± 1.14	7.66 ± 0.74	9.95 ± 0.39	2.13 ± 0.11
21:0	0.09 ± 0.01	0.12 ± 0.02	n.d	n.d	n.d	n.d	0.05 ± 0.02
22:0	n.d	n.d	1.31 ± 0.21	1.39 ± 0.26	0.12 ± 0.02	0.12 ± 0.01	0.11 ± 0.02
22:1*n*-11	n.d	n.d	n.d	n.d	n.d	n.d	1.94 ± 0.20
22:1*n*-9	n.d	n.d	n.d	n.d	0.37 ± 0.03	0.35 ± 0.05	0.29 ± 0.05
22:4	0.24 ± 0.04	0.41 ± 0.05	n.d	n.d	n.d	n.d	n.d
22:4	0.13 ± 0.02	0.29 ± 0.04	n.d	n.d	n.d	n.d	n.d
22:5*n*-6	0.31 ± 0.05	0.76 ± 0.12	n.d	n.d	n.d	n.d	n.d
22:5*n*-3	1.07 ± 0.10	1.03 ± 0.07	2.38 ± 0.31	3.64 ± 0.47	n.d	n.d	0.69 ± 0.06
unknown	n.d	0.58 ± 0.23	n.d	n.d	n.d	n.d	n.d
22:6*n*-3	8.75 ± 1.00	11.85 ± 1.01	n.d	n.d	n.d	n.d	4.59 ± 0.32
24:0	n.d	n.d	n.d	n.d	0.33 ± 0.05	0.31 ± 0.03	n.d
24:1*n*-9	n.d	n.d	n.d	n.d	0.62 ± 0.05	0.72 ± 0.07	n.d
∑*n*-3	32.03 ± 3.62	40.07 ± 1.54	27.35 ± 1.87	27.61 ± 2.30	19.16 ± 1.54	25.24 ± 1.42	11.43 ± 0.51
∑*n*-6	9.02 ± 1.25	6.94 ± 0.46	5.00 ± 0.24	3.45 ± 0.44	22.42 ± 1.59	24.18 ± 0.07	18.09 ± 0.23
∑*n*-3/∑*n*-6	3.66 ± 0.98	5.79 ± 0.37	5.46 ± 0.25	8.04 ± 0.36	0.85 ± 0.03	1.04 ± 0.06	0.63 ± 0.03
∑BCFA	4.59 ± 0.53	5.48 ± 0.33	n.d	n.d	n.d	n.d	n.d
∑SFA	19.52 ± 2.36	22.39 ±1.00	46.30 ± 3.35	48.78 ± 3.37	29.35 ± 3.48	26.02 ± 0.50	25.72 ± 1.42
∑MUFA	32.99 ± 0.92	19.95 ± 1.39	20.88 ± 1.62	20.07 ± 1.66	29.07 ± 2.32	24.42 ± 1.48	44.77 ± 0.81
∑PUFA	42.81 ± 2.65	48.48 ± 1.80	32.82 ± 1.94	31.19 ± 2.73	41.58 ± 3.08	49.43 ± 1.42	29.52 ± 0.64

Table SI2: Summary of the environmental parameters measured at the time of sampling in locations with *versus* without the influence of organic-rich waters from fish farming activities (+Org or -Org, respectively). Values are expressed as a percentage and are averages of three replicates (n=6) ± SD.

**Oxygen (mg/L)**		**Salinity**		**Temperature (ºC)**		**pH**	
**+Org**	**-Org**	**+Org**	**-Org**	**+Org**	**-Org**	**+Org**	**-Org**
10.1	15.9	33.9	25.7	16.7	16.4	8.10	9.14
9.9	12.2	33.8	25.5	16.5	17.1	8.16	9.06
8.3	13.2	33.2	33.5	15.4	17.0	8.20	8.95
8.3	9.4	33.8	25.6	15.4	16.9	8.22	9.07
13.6	13.0	33.2	33.5	16.7	17.2	8.58	9.29
12.7	12.7	33.9	25.8	16.6	17.0	8.61	9.10
Average ± SD		Average ± SD		Average ± SD		Average ± SD	
10.5 ± 2.2	12.7 ± 2.1	33.6 ± 0.3	28.3 ± 4.1	16.2 ± 0.6	16.9 ± 0.3	8.3 ± 0.2	9.1 ± 0.1

Table SI3: Nutritional compostion of the formulated fish feed provided to the fish at the fish farming location (+Org).

SORGAL, SOCIEDADE DE ÓLEOS E RAÇÕES, SA

Standard orange 4

Seabass; Gilthead Seabream; Complete Animal Feed; From 50g to 150g

Analytical Constituents

Crude Protein 43.0%; Crude Fat 17.0%; Crude Ash 10.0%; Crude Fiber 3.0%; Calcium 1.9%; Phosphorus 1.4%; Sodium 0.4%

Additives (per kg of feed)

Vitamins, pro-vitamins and chemically well-defined substances having similar affect:

3a672a - Vitamin A 5.000 UL; E671 - Vitamin D3 1.000 UL; 3a700 – All rac alfa-tocoferyl acetate 200 UL; 3a312 – Vitamin C (Ascorbil Monophospahte) 100 mg;

Compounds of trace elements

E1 – Fe - ferrous chelate of Glycine 2.0 mg; **3b202 – I –** Calcium iodate anhydrous 1.1 mg; **E4 – Cu** – Cupric chelate of amino acids, hydrate 12.0 mg; **E5 – Mn** – Manganese and amino acids chelate, hydrate 32.0 mg; **3b606 – Zn** – Zinc and amino acids chelate, hydrate 48.0 mg; **3b815 – Se** – L-Selenomethionine 0.1 mg

Amino acids, their salts and analogues

3.2.2 - L – Lysine monohydrochloride 2.5 g; **3a301** – DL Methionine 3.0 g

Antioxidants

E310 – 0.39 mg; **E320 + E321** – 100 mg

Anticaking agent

E562 – 395 mg; **1m558** – 382 mg

Ingredients

10.4.2 Fish meal; **1.11.1** Wheat; **9.4.1** Processed animal protein (non ruminants)

9.11.1 Feather meal; **2.18.4** Soy (bean) meal, dehulled; **10.4.6** Fish Oil; **2.19.4** Sunflower seed meal, dehulled; **3.7.4** Horse bean, dehulled; **9.7.1** Non ruminant blood meal; **9.2.1** Poultry fat; **10.4.4** Fish protein, hydrolysed; **1.2.8** Corn gluten meal; **12.1.4** Bacterial protein from *Corynebacterium glutamicum*; **11.3.3** Monocalcium phosphate; **12.1.5** Brewer's yeast; **12.2** Other fermentation by-products

Contains fish meal, non ruminant blood products and processed animal products – not to be used with ruminants. Not to be used with farming animals, except aquaculture feeds and fur animals.

Store cool and dry, away from direct sunlight
